# Isolation, Characterization, and Insecticidal Activity of an Endophyte of Drunken Horse Grass, *Achnatherum inebrians*

**DOI:** 10.1673/031.013.15101

**Published:** 2013-12-13

**Authors:** YingWu Shi, Xuebing Zhang, Kai Lou

**Affiliations:** Institute of Microbiology, Xinjiang Academy of Agricultural Sciences, Urumqi, China, 830091

**Keywords:** cotton aphid, endophytic microorganisms, microbial insecticide

## Abstract

Endophytic microorganisms reside within plant tissues and have often been found to promote plant growth. In this study, endophytic microorganisms were isolated from the roots, stems, leaves, and seeds of healthy drunken horse grass, *Achnatherum inebrians* (Hance) Keng (Poales: Poaceae), through the use of a grinding separation method and identified by a dual approach of morphological and physiological observation and 16S rRNA gene-based (for bacteria) and internal transcribed sequence-based (for fungi) molecular identification. The endophytes were then inoculated into liquid media for fermentation, and their crude extracts were employed for insecticidal activity tests using slide disc immersion and nebulization methods. A total of 89 bacteria species, which were classified into eight genera, *Bacillus, Pseudomonas, Actinomyces, Corynebacterium, Acinetobacter, Sphingomonas, Paenibacillus*, and *Phyllobacterium,* and two fungi, *Claviceps* and *Chaetomium,* were isolated. Of these species, isolates *Streptomyces albus* (Rossi-Doria) Waksman and Henrici (Actinomycetales: Streptomycetaceae) (GA) and *Claviceps purpurea* (Fr.) Tul. (Hypocreales: Clavicipitaceae) (PF-2) were shown to produce mortality rates of more than 90% in the cotton aphid, *Aphis gossypii* Glover (Hemiptera: Aphididae), after first and second screenings. The isolates PF-2 and GA associated with *A. inebrians* had significant insecticidal activities towards *A. gossypii* Glover (Hemiptera: Aphididae) and may provide a new biological resource for exploring a new microbial insecticide.

## Introduction

Drunken horse grass, *Achnatherum inebrians* (Hance) Keng (Poales: Poaceae), is an important perennial bunchgrass in China associated with the narcosis of grazing animals by endophyte infection (Li et al. 2009; de Oliveira et al. 2010). It is an intoxicating perennial bunchgrass found mainly in north and northwestern China in the grasslands of Gansu, Xinjiang, Qinghai, Inner Mongolia, and Tibet (Zhang et al. 2009). *Achnatherum inebrians* usually grows on roadsides, gully slopes, and even in the harsh conditions of the alpine or subalpine grasslands in the Qinghai-Tibetan, Tianshan, and Qilian mountains (Li et al. 2009; Linaldeddu et al. 2011), where it has caused grassland degradation and environmental deterioration (Li et al. 2009; Zhang et al. 2010; Linaldeddu et al. 2011). Recent studies have highlighted its potential for expansion and its impact on other communities (Miles et al. 1996). It has become an economic problem and a threat to the conservation of natural systems.

Endophytic microorganisms are present in various plant species and rarely produce any disease symptoms (Borneman et al. 2000; Hanada et al. 2010; Teng et al. 2010; Wang et al. 2010; Bae et al. 2011; de Siqueira et al. 2011). The asymptomatic internal colonization of healthy plant tissue by microorganisms is a widespread and well-documented phenomenon. “Endophyte” is an all-encompassing topographical term that includes all organisms that have a variable period of their life cycle during which they colonize the living internal tissues of their hosts without producing the symptoms of disease (Matthews et al. 1990; Li et al. 2003; Yanni et al. 2011). Common endophytes include a variety of bacteria, fungi, and actinomycetes, and they can be isolated from wild (Shi 1997; Teng et al. 2010; Melnick et al. 2011) or cultivated crops (Jukes et al. 1969; Pedrosa et al. 2011; Yang et al. 2011) of either the monocotyledonous (Woese 1987; Jha and Kuma 2009) or dicotyledonous plant groups (Miles 1996; Chen 2008).

Endophytes occupy microniches within plant tissues and some have been found to be growth-promoting endophytes (Kumar et al. 2001; Zhou et al. 2010; Senthilkumar et al. 2011). Some endophytes show resistance to abiotic and biotic stresses (Saitou and Nei 1987; Ardanov et al. 2011). These endosymbionts enhance the absorption of nutrients by the host plant, which leads to improved vegetative growth. Endophytes provide *A. inebrians* with a strong competitive ability due to an increased host tolerance to drought (Li et al. 2004b), salt (Gou 2007), cold (Chen 2008), and pathogenic fungi (Li et al. 2004a, b). The presence of endophytic fungi in healthy *A. inebrians* has been demonstrated (Li et al. 2009; Linaldeddu et al. 2011).

Despite the advantages of endophytes, they can have economic costs, as some cause problems in livestock throughout the world. *Neotyphodium coenophialum* causes tall fescue toxicosis (Bacon et al. 1977) and *Neotyphodium lolii* causes ryegrass staggers (Fletcher and Harvey 1981). In China, *Neotyphodium gansuense* is symbiotic with *A. inebrians* (Li et al. 2004b) and inhibited the growth of three fungal pathogens (Li et al. 2007). Endophytic *Penicillium* sp. with insecticidal activities against the diamond backed moth, *Plutella xylostella*, and the mustard aphid, *Lipaphis erysimi*, was screened from the fresh roots of *Derris elliptica* (Hu et al. 2008).

Little is known about the endophytic bacteria and pesticidal microorganisms that colonize *A. inebrians* during the growing season. It is unclear whether these endophytic microorganisms affect *Aphis gossypii* Glover (Hemiptera: Aphididae) in the crops in Xinjiang. In this study, the species and quantitative changes produced by endophytic microorganisms and their insecticidal activities were studied. The results will provide theoretical guidance for discovering and using microorganisms to control *A. gossypii* and thus increase crop yields.

## Materials and Methods

### Sampling of plant materials

*Achnatherum inebrians* was collected in its native habitat from a mountain near Urumuqi City in Xinjiang Province, China (26° 30′ 24.1″ N, 106° 27′ 43.3″ E) in May-July 2008. The samples were taken to the laboratory together with the soil and replanted for further experiments.

### Isolation of endophytic microorganisms

Fresh, healthy *A. inebrians* plants were washed thoroughly with tap water to remove adhering soil and debris, immersed in 70% ethanol for 3 min, washed with fresh sodium hypochlorite solution (2.6% available CF) for 5 min, and finally rinsed 3 times in sterile distilled water. Surface-disinfected samples were then rinsed 10 times (5 min each rinse) in sterile phosphate buffer. To confirm that the surface disinfection process was successful and to verify that no biological contamination from the surface of the beet was transmitted into the root tissues during maceration, sterility checks were conducted for each sample to monitor the effectiveness of the disinfection procedures. For these checks, sample impressions were taken and 0.1 mL from the final rinse was plated out onto Petri plates of tryptic soy agar, potato dextrose agar, and Gause's No. 1 synthetic medium agar. The absence of bacteria and fungi after 6 days of incubation during the sterility checks was taken to confirm sterility and confirm the isolated microbes were endophytic.

### PCR amplification and sequencing of bacterial 16S DNA and fungal internal transcribed spacer

The total genomic DNA of isolates was extracted and amplified as described earlier (Hanada et al. 2010). Amplification of the 16S rRNA gene of endophytic bacteria was performed using universal primer set pA (5-AGAGTTTGATCCTGGCTCAG-3) and pB (5-AAGGAGGTGATC C AGC CGC A-3) (Woese 1987; Stackebrandt et al. 1994). Primer pair TS1 (5-TCCGTAGGTGAACCTGCGG-3) and ITS4 (5-TCCTCCGCTTATTGATATGC-3) (Borneman, et al. 2000) were used for the amplification of the fungal ribosomal DNA internal transcribed spacer regions 1 and 2 of all isolates. The PCR products were purified using a DNA gel extraction kit (SanPrep Column PCR Product Purification Kit) and cloned into pMD18-T vector followed by sequencing. Sequence analysis was performed using the BLAST algorithm (www.ncbi.nlm.nih.gov). An evolutionary distance matrix was generated as described by Jukes and Cantor (2010). The evolutionary tree for the dataset was inferred from the neighbor-joining method of Saitou and Nei (1987) using the neighbor-joining program of MEGA version 2.1 (Kumar et al. 2001).

### Screening of pesticidal microorganisms and bioassays of insecticidal activities

Endophyte cultures were grown in a liquid medium (soluble starch (20 g), soy flour (15 g), yeast powder (5 g), protein peptone (2 g), calcium carbonate (4 g), sodium chloride (4 g), pH 7.0–7.2). After 8 days of incubation at 25° C on a rotary incubator at 120 rotations per minute (75–85% RH, 12:12 L:D), the supernatants were filtered through 0.22 µtn pore size filters to remove bacterial and fungal mycelia and spores. The different components were separated out from of the endophytic microbial metabolites by solvent extraction, and insecticidal activity of different components was tested. The filters were extracted 3 times by using 4 successive solvents of increasing polarity, benzene, ether, chloroform, and 95% ethyl alcohol, distilled at 65° C for 3 hr. The residues obtained after solvent evaporation (at the reduced pressure) were indexed as follows: E1 for the benzene extract, E2 for the ether extract, E3 for the chloroform extract, and E4 for the 95% ethyl alcohol extract. The extracts of the tested plants were then dissolved in the appropriate solvent at a concentration of 1% and kept at 4° C.

The filtrates were then used for insecticidal testing. The test insect, *A. gossypii*, was grown in a greenhouse (Institute of Plant Protection, Xinjiang Academy of Agricultural Sciences). Endophyte cultures were tested for their insecticidal activities using slide disc immersion and nebulization methods. The leaf dipping method and potter spraying method were used to test the bioactivity of each fraction against aphids under laboratory conditions. A mixture of acetone and water (1:1) was used to dilute the tested extracts. In the leaf dipping method, a cabbage leaf was dipped in the test solutions for 20 sec, and then 1000 aphids were added on its surface. It was then transferred into Petri dishes (9 cm in diameter) that had moist filter paper in the bottom. The potter spraying method was conducted by spraying test solutions (1 mL for each test) on 1000 aphids in the potter spraying tower, and then 1000 aphids were added on each side of the cabbage leaf. The leaf with 1000 aphids was transferred into Petri dishes with moist filter paper in the bottom. The control was treated with a mixture of solvent and water only. The following 5 concentration levels were tested: 0.02, 0.05, 0.1, 0.5, 1.0 (volume fraction). Each concentration was replicated 3 times. The aphids were maintained in the insectory at 28 ± 1° C, 75 ± 2% RH, and 14:10 L:D conditions. The number of living and dead aphids was recorded after 24 and 48 hr, and mortality was corrected using Abbott's formula (Fleming et al 1985). The LC50 value of each treatment was determined by using probit analysis with the SPSS program PASW Statistics 18 (IBM, http://www01.ibm.com/software/analytics/spss/). The data were subjected to statistical analysis with Duncan's multiple range test at *p =* 0.05 to test the differences among the treatments.

### Physiological and biochemical characterization

Morphology and gram type were determined using a trinocular phase contrast fluorescent microscope (Olympus AX 80T, www.olympus-global.com). Bacterial motility was tested by growth in a semisolid 0.3% mannitol motility test medium. Oxidase and catalase were determined using commercially available disc tests (HiMedia, www.himedialabs.com). Utilization of carbon sources was examined using the Vitek Auto-Microbic system (bioMerieux, www.biomerieux-usa.com). The Vitek test was repeated twice. Isolates were characterized by Vitek AMS, colony morphology, catalase production, oxidase test, and gram stain (Matthews et al. 1990). All isolates were tested 3 times using the Vitek test, according to the manufacturer's recommendations, with reactions observed after 24 hr.

## Results

### Isolation and identification of endophytes

**Figure 1.**
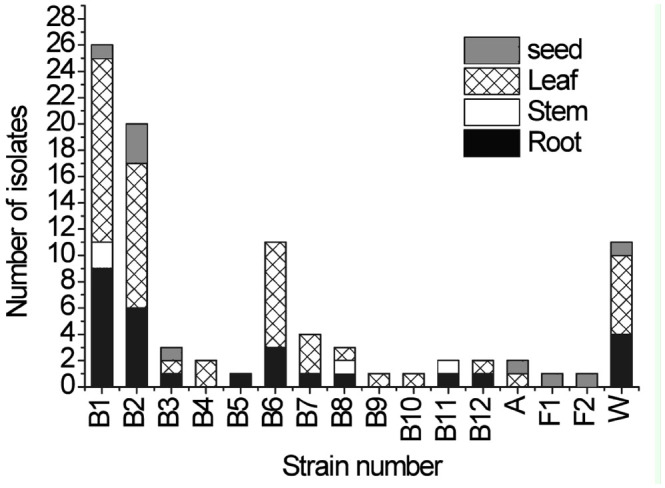
The distribution of endophytes in different parts of *Achnatherum inebrians.* B1–B12: bacteria; A: actinomycete; F1–F2: fungus; W; no identification. High quality figures are available online.

During this investigation, 121 *A. inebrians* root segments, 60 leaf segments, and 33 seed segments were incubated, and 89 bacterial isolates and 2 fungal isolates were obtained. From all the isolates, 9 bacterial species, 2 fungal species, and 1 actinomycete species were identified (Figure 1). The 2 fungal isolates were characterized as *Claviceps purpurea* (Fr.) Tul. (Hypocreales: Clavicipitaceae) and *Chaetomium globosum* Kunze ex Fr. (Sordariales: Chaetomiaceae). The endophytic actinomycete isolate was characterized as *Streptomyces rochei* Berger et al. (Actinomycetales: Streptomycetaceae). At the genus level of endophytes, *Bacillus subtilis* (Ehrenberg) Cohn (Bacillales: Bacillaceae) was the bacterium most frequently isolated in the *A. inebrians* and accounted for 58.4% of the total number of endophytic bacteria. Species of endophytic bacteria were most abundant in leaf tissues.

### The screening of insecticidal activity of isolates

The insecticidal activities of the 91 bacterial and fungal isolates were tested using *A. gossypii.* A total of 86 isolates displayed some insecticidal activity towards the aphid, while only 5 isolates (GA, PF-2, 2N153, 2N185, and 2P118) had high activity toward the aphid (Table 1). Seventy-five isolates caused aphid mortality rates of 60% or more, and 10 isolates caused aphid mortality rates of 40% or less. For screening insecticidal activities, the 5 virulent isolates were tested 48 hr after the initial observation results (Table 1). Isolates GA and PF-2 were found to display the highest insecticidal activities, highest rates of mortality, and best efficiency. Isolates GA and PF-2 were selected from *A. inebrians* and classified in the genera *Streptomyces* and *Claviceps,* respectively.

**Figure 2.**
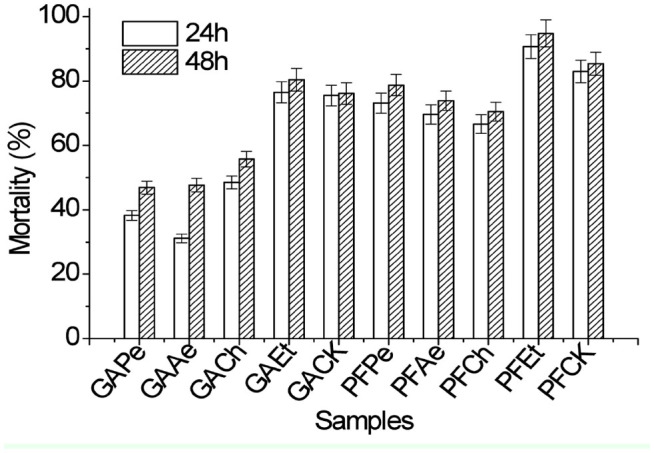
Insecticidal activities of 4 isolates against *Aphis gossypii.* GAPe, GAAe, GACh, GAEt, PFPe, PFAe, PFCh, and PFEt represent the extract of isolates G A and PF-2 after the fermentation of petroleum ether, ethyl ether, chloroform, and ethanol, respectively. GACK and PFCK represent fermentation broth of isolates GA and PF-2course, respectively. Data points are a mean of 1000 insects for each treatment ± SE. High quality figures are available online.

### Insecticidal activity of fermentation extracts

The insecticidal activities of 4 fermentation extracts of isolates GA and PF-2 towards *A. gossypii* were examined using the dipping method (Figure 2). The insecticidal activities of the 4 extracts of isolates GA and PF-2 after the fermentation of petroleum ether, ethyl ether, chloroform, and ethanol were 46.89%, 47.67%, 55.75%, 80.40% and 78.71%, 73.87%, 70.45%, 94.82%, respectively. The results from the initial tests indicated that the toxic component of the fermentation was being concentrated in the hydrophilic portion of the fermentation liquid, namely the ethanol extract. However, there were significant differences between the tested extracts. Strong insecticidal activities (80.4% and 94.8%) were obtained from the ethanol extracts of isolates GA and PF-2, respectively. Weak insecticidal activities (80–93%) were obtained from the petroleum ether, ethyl ether, and chloroform extracts of isolates GA and PF-2. The other 6 extracts showed significantly lower insecticidal activities towards the cotton aphid after the 48 hr treatment.

A mathematical model for the mortality of the aphid and the extract concentration and the main factor affecting the concentration of the extracts was established (Table 2). The linear regression equations of GA and PF-2 were respectively:

y = 5.9460 + 0.5379x

(LC_50_ = 0.0174 mg/mL, 95% confidence limit of 0.0047–0.0286, F = 50.2084 > F_0.05_ = 0.0058, R^2^ = 0.94362)

and

y = 6.6499 + 0.7095x

(LC_50_ = 0.0047 mg/mL, 95% confidence limit of 0.0005–0.0113, F = 50.2084 > F_0.05_ = 0.0058, R^2^ = 0.94362)

### Morphological characteristics of isolates GA and PF-2

**Figure 3.**
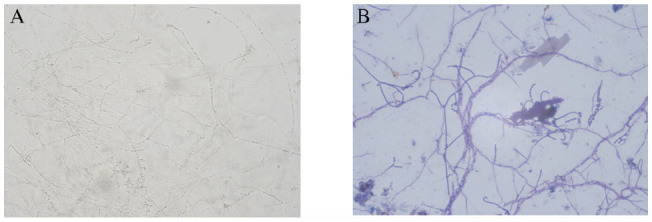
Shape of the spore producing chains of the isolates. A: isolate PF-2; B: isolate GA. High quality figures are available online.

Based on microscopic observations, isolate GA was an elongated rod-shaped bacterium in its spore. Isolate PF-2 was smaller than GA, with mycelia, brown-colored, branching, and a diaphragm. It has conidial stalks that do not branch and that are also brown in color. All bud-type cells produced conidia during sporulation but were more solitary. However, the cells also clustered as spores with an elliptical shape (Figure 3). On different media, isolate GA produced gas, its substrate mycelium changed slightly, and it also produced a soluble pigment. Isolate PF-2 produced no obvious changes in aerial or substrate mycelium, and no soluble pigment was produced (Table 3).

### Physiological and biochemical characteristics of GA and PF-2 isolates

The physiological and biochemical characteristics of the isolates are shown in Table 4. Isolate GA utilized carbon and nitrogen sources other than aspartate. Isolate GA liquified gelatin and coagulated milk but did not hydrolyze starch or fat. Isolate GA produced hydrogen sulfide, melanin, and urease, and its methyl red and V-P reactions were negative. Isolate GA could not utilize citrate. Isolate PF-2 liquified gelatin and coagulated milk but did not hydrolyze either starch or fat. Isolate PF-2 did not produce hydrogen sulfide or melanin but produced urease and indole. Its V-P reaction was positive, and its methyl red reaction was negative. Isolate PF-2 could utilize citrate. Isolates GA and PF-2 were not sensitive to ampicillin. Strain GA had broad temperature (25–50° C) and pH (5–10) ranges in Gao-1 broth medium, and its optimal growth temperature was 37° C. Its NaCl tolerance was up to 9%. Isolate GA was motile, with a growth temperature range of 4–41° C. Isolate PF-2 had a broad temperature range (25–45° C) and pH 7–8 in potato dextrose agar broth medium, and its optimal growth temperature was 28° C. Its NaCl tolerance was up to 8%.

## Discussion

Most isolated endophytic fungi are primarily fungal anamorphs that belong to the *Deutero-mycotina,* including several hyphomycetes, whereas *Ascomycotina* are comparatively scarce. *Neotyphodium* is an endophyte of *A. inebrians.* It was described by Li et al. (2004a, b) and subsequently characterized (Li et al. 2003). *Neotyphodium* was not isolated in this study. At the genus level, *B. subtilis* is the most frequently isolated endophyte and accounts for 58.4% of the total number of endophytic bacteria. Species of endophytic bacteria are most abundant in the leaf tissues of *A. inebrians.*

The species isolated in this study may be classified into three groups: 1) economically important insecticidal and antimicrobial microorganisms, i.e., *C. purpurea, C. globosum, Pseudomonas fluorescens* (Flügge) Migula (Pseudomonadales: Pseudomonadaceae), and *Paenibacillus polymyxa* (Prazmowski) Ash et al. (Bacillales: Paenibacillaceae); 2) common and abundant bacteria that are not considered pathogens, i.e., *B. subtilis* and *Bacillus pumilus* (Bacillales: Bacillaceae); and 3) species that are occasionally present in *A. inebrians,* i.e., *Bacillus cereus* Frankland and Frankland and *Stenotrophomonas maltophilia.* These microorganisms are present at intermediate or low frequencies. Further studies are needed to firmly establish if endophytes are the result of a mutualistic relationship with the host *(A. inebrians*) or competitive colonization.

Endophytes, as a plant micro-ecological system, have the ecological significance of enhancing the host plant's accommodative ability to the environment. Endophytic microorganisms are a poorly exploited source of insecticidal factors, which could be used for the development of new natural insecticides and represent a potential new field of study. Research shows that there are many endophytes within the organization of *A. inebrians* and that the vast majority of endophytes are bacterial populations. The results of our study are consistent with this earlier research.

Our study found that the varieties and quantities of endophytic microorganisms varied greatly within different tissues of *A. inebrians.* Endophytes were mainly located in the leaves > root > seeds > stems. There may also have been endogenous bacteria on the host as a result of selective and adaptive responses. Thus, the selection of endophytes on a host may be possible.

Insecticidal activity tests indicated that 4 organic solvent extracts from fermentation of isolates GA and PF-2 were the most toxic towards *A. gossypii.* Crude extracts of these fermentations contained chemical compositions with insecticidal activities. The specific compounds that accounted for the insecticidal activities require further isolation and purification. The results from this research indicate that the crude fermentation extracts of isolates GA and PF-2 may contain compounds that are effective insecticides. However, the insecticidal spectra of these compounds are not clear and require further study.

The fermentation extracts of isolates GA and PF-2 were the most toxic toward *A. gossypii,* suggesting that they are a special type of aphicide. Therefore, through the use of an endophyte preparation process to produce a pesticide from a microbial source, suitable ways to enhance the effectiveness and efficiency of these extracts might be found for a new and efficient pesticide. Environmental protection is important, and the development of endophyte-derived pesticides could enlarge the comprehensive utilization of resources such *A. inebrians.* Thus, the biological pesticide industry might have prospects for further development. The compound structure and anabolic mechanism of the insecticidal ingredients and safety evaluations require further study.

**Table 1.**
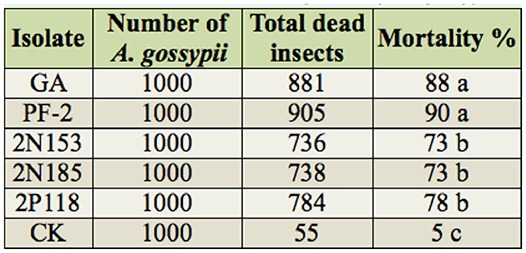
The endophyte of *Achnatherum inebrians* insecticidal activities against *Aphis gossypii.*

**Table 2.**
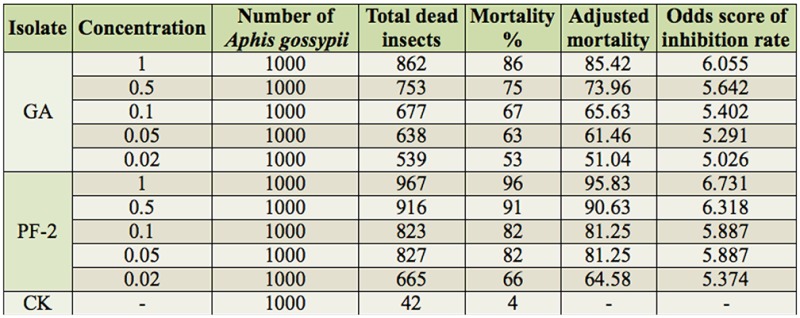
Isolates GA and PF-2 insecticidal activities against *Aphis gossypii.*

**Table 3.**
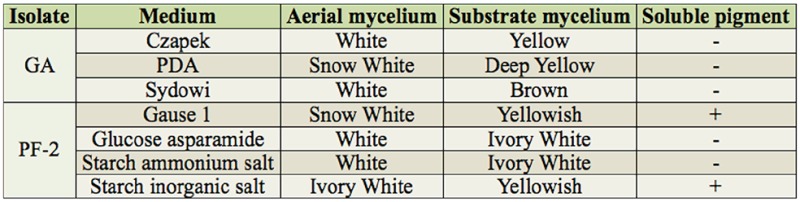
Cultural characteristics of the isolates GA and PF-2 in different mediums.

**Table 4.**
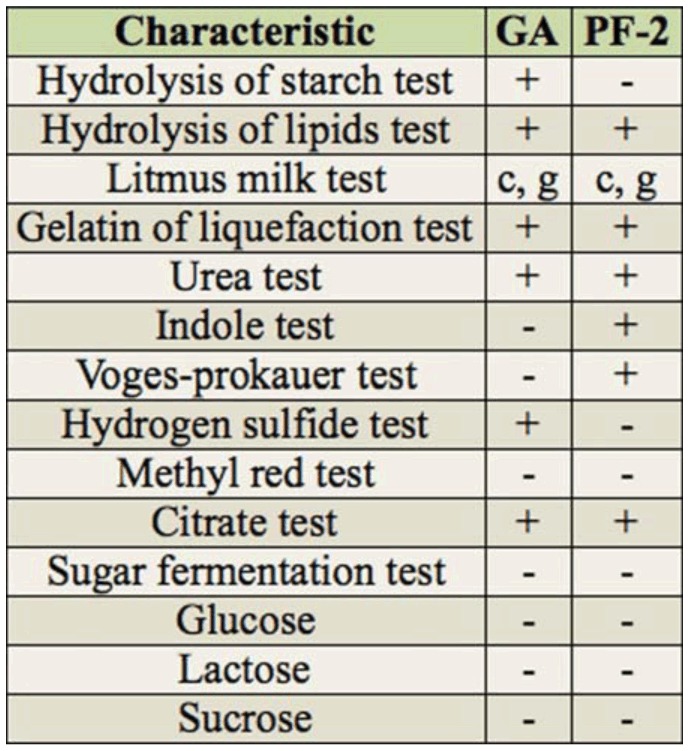
Physiological and biochemical characteristics of the isolates PF-2 and G A.
